# Endometrial osseous metaplasia: Clinicopathological study of a case and literature review

**DOI:** 10.4103/0974-1208.69329

**Published:** 2010

**Authors:** T Umashankar, Shobhana Patted, RS Handigund

**Affiliations:** Department of Pathology, Yenepoya Medical College, Mangalore, India; 1 Department of Obstetrics and Gynecology, JN Medical College, Belgaum, India; 2 Department of Pathology, KLES Dr. Prabhakar Kore Hospital and MRC, Belgaum, India

**Keywords:** Osseous metaplasia, endometrium, hysteroscopy

## Abstract

Endometrial osseous metaplasia is an uncommon clinical entity with the presence of bone in the endometrium. Most of the cases clinically present with secondary infertility following an abortion. Various theories have been proposed and the most accepted theory is metaplasia of the stromal cells into osteoblastic cells that produce the bone. It is important to distinguish this condition from the mixed mullerian tumor of the endometrium to avoid hysterectomy. Removal of these bony bits leads to spontaneous conception. We present one such case in a 25-year-old female patient presented with secondary infertility.

## INTRODUCTION

Endometrial osseous metaplasia is a rare clinical entity with the presence of mature or immature bone in the endometrium. Nearly 80 cases have been reported in the world literature including around nine cases from India. In most of the reported cases, ossification was followed by an abortion and patients presented with infertility.[1–5]

Various theories have been proposed and the accepted theory is metaplasia of the stromal cells into osteoblastic cells that produce mature bone.[3,4,6] Hysteroscopic evacuation of these bony spicules is the preferred management[7,8] and most of the patients conceived after the evacuation.[2,5,7]

## CASE REPORT

A 25-year-old female patient presented to gynecology outpatient department with the history of infertility. She was married for 3 years. Past history revealed that before marriage she had conceived and underwent medical termination of pregnancy by dilatation and curettage at 8 weeks of gestation. Following the event, her menstrual history was normal. She did not have any other significant complaints.

The couple was subjected for infertility workup. The semen analysis of the husband was normal. Evaluation of the female partner by transvaginal ultrasonography revealed an echogenic area in the uterine cavity measuring 1 cm×1 cm suggestive of retained products of conception. The patient was subjected for a diagnostic hysteroscopy.

Hysteroscopy was done using rigid hysteroscope (Karl Storz) with saline as distending media. Endometrial cavity revealed multiple small, hard bony spicules, which were removed using hysteroscopic forceps and submitted for histopathological study.

Grossly the bits were multiple, small, bony in texture, and totally measured about 2 cm×1 cm in dimension. Multiple scanty soft tissue bits were included in the biopsy material. The bony bits were kept for decalcification.

The hematoxylin and eosin stained paraffin sections showed trabeculae of woven bone with non-hematopoietic bone marrow. Endometrial tissue was scanty and predominantly composed of tubular glands with scanty stroma. No secretory activity was noted in the endometrial glands. Inflammatory reaction, necrosis, and products of conception were absent. No evidence of granuloma was seen.

The serum calcium was 9.2 mg/dl and phosphorus was 3.7 mg/dl. Both were within the normal range.

**Figure 1:**
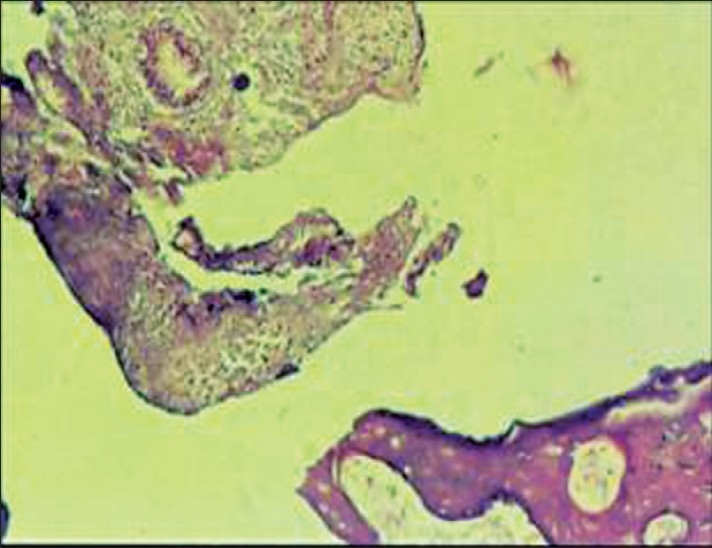
Photomicrograph showing endometrial glands and stroma with spicules of woven bone (H and E, ×200)

A histological diagnosis of osseous metaplasia of endometrium was made [Figure 1].

Further evaluation could not be done as the patient was lost for follow up.

## DISCUSSION

Ossification of the endometrium is an uncommon clinical entity. It is also described by various other names such as endometrial ossification, ectopic intrauterine bone, and heterotopic intrauterine bone.[1,2,9] Ossification is also reported in the cervix,[3,10] the ovary,[11] and the vagina.[12]

In most of the reported cases, the osseous changes in the endometrium was followed by a previous history of abortion.[1,2,3,7] Majority of the patients are in the reproductive age group with history of first trimester abortion either therapeutic or spontaneous and have normal menstrual cycle in the post-abortive period as noted in our case. The time interval between the antecedent abortion and discovery of endometrial ossification varies from 8 weeks to 14 years in reproductive age group.[7] Shimazu and Nakayama[9] described endometrial ossification in a 62-year-old post-menopausal woman who also had the history of abortion 37 years earlier to the diagnosis of endometrial ossification.

Common clinical presentations are menstrual irregularities, pelvic pain, dyspareunia, vaginal discharge, and secondary infertility.[9] Bhatia and Hoshiko[3] incidentally discovered endometrial and cervical osseous metaplasia in a 24-year-old woman. Shroff *et al*.[4] described a case of endometrial ossification in a woman presenting with primary infertility. In our case secondary infertility was the only complaint.

Osseous metaplasia of endometrium is described as an endogenous non-neoplastic pathological condition as no tissue reaction is found in the endometrial tissue studied and the endometrium showed normal regular cyclical changes as noticed in the present case.[1,3] Various theories have been described in the published literature in the pathogenesis of endometrial osseous metaplasia.

Heterotopia, dystrophic calcifications, and ossification of post-abortive endometritis, metastatic calcification, metaplasia in healing tissue, prolonged estrogenic therapy after abortion, and retained fetal bone are the common proposed theories.[1–3,5,6]

Majority of the patients had first trimester abortion and it is unlikely that the endometrial bone is of fetal origin and there was no fetal tissue found in the biopsy material studied. Biopsy also showed minimal or no tissue reaction so as to explain ossification was dystrophic in nature.

Most of the cases reported did not have any evidence of hypercalcemia or the conditions leading to hypercalcemia to support the theory of metastatic calcification. Adamson and Sommers[13] reported a case of endometrial ossification in a patient who was taking high dose of calcium and vitamin D for long term.

In India, endometrial tuberculosis should be ruled out as it can cause infertility as well as calcification and subsequent ossification.[4]

Most of the authors opine that the bone formed is as a result of the metaplasia of endometrial stromal cells into osteoblastic cells that produce bone in the endometrium.[3,4,9]

Bahceci and Demirel[7] suggested that post-abortive chronic endometritis stimulates the release of superoxide radicals and tumor necrosis factor from the inflammatory cells. Long-term exposure of superoxide radicals and tumor necrosis factor on multipotent stromal cells in patients with deficient superoxide dismutase activity in the endometrium leads to metaplasia of the stromal cells into osteoblastic cells.

Chronic endometritis also stimulates the proliferation of mesenchymal cells that have inherent property of metaplasia and can differentiate into chondroblasts or osteoblasts.[4]

Cayuela *et al*.[14] studied DNA pattern in a 27-year-old woman who was diagnosed to have endometrial osseous metaplasia following first trimester abortion. They found same DNA pattern in the blood of the patient and in the endometrial biopsy including the bone removed from the endometrium. No genetic material of male or fetus was found. They opined that pluripotent mesenchymal cells, mϋllerian cells, and fibroblasts undergo osteoblastic metaplasia in response to inflammation or curettage supporting the osseous metaplasia theory.

It is important for the pathologists to recognize the nonneoplastic nature of this condition to avoid making a wrong diagnosis of malignant mϋllerian tumor of the uterus.[3,7,9]

Initial studies recommended a series of dilatation and curettage to remove the bone from the endometrium. Vigorous single curettage should be avoided which may lead to synechiae formation.[3] Recent studies recommend hysteroscopic removal of the bone under the ultrasonic guidance that helps proper visualization and complete removal of the bony spicules that may be embedded in the myometrium.[7,8,14]

Use of estrogen is controversial as it can promote osteogenesis and can be one of the causes of endometrial ossification.[3] In a woman with normal regular menstrual cycle, endogenous hormones are sufficient for endometrial regeneration.[7]

Bone in the endometrium can act as an intrauterine contraceptive device and its complete removal can restore the fertility and spontaneous conception.[2,5,7]

## CONCLUSION

Endometrial ossification is a rare but treatable cause of infertility in which intrauterine bone prevents normal conception. Chronic endometritis evokes metaplastic changes in the pluripotent endometrial stromal cells into osteoblastic cells that lay down bone. Surgical pathologists should be aware of the condition to avoid making erroneous diagnosis of malignant mixed mϋllerian tumor of endometrium. Complete removal of the bony spicules from the endometrial cavity by hysteroscopy under ultrasonic guidance regains the fertility
